# The evolution of morality and its rollback

**DOI:** 10.1007/s40656-018-0190-5

**Published:** 2018-03-21

**Authors:** Brian Garvey

**Affiliations:** Department of Politics, Philosophy and Religion, Lancaster University, County South, Bailrigg, Lancashire LA1 4YL UK

**Keywords:** Evolutionary psychology, Evolution of morality, Cognitive modularity, Evolutionary rollback, Embedded cognition

## Abstract

According to most Evolutionary Psychologists, human moral attitudes are rooted in cognitive modules that evolved in the Stone Age to solve problems of social interaction. A crucial component of their view is that such cognitive modules remain unchanged since the Stone Age, and I question that here. I appeal to evolutionary rollback, the phenomenon where an organ becomes non-functional and eventually atrophies or disappears—e.g. cave-dwelling fish losing their eyes. I argue that even if cognitive modules evolved in the Stone Age to solve problems of social interaction, conditions since then have favoured rollback of those modules. This is because there are institutions that solve those problems—e.g. legal systems. Moreover, evidence suggests that where external resources are available to perform cognitive tasks, humans often use them instead of internal ones. In arguing that Stone Age cognitive modules are unchanged, Evolutionary Psychologists say that evolutionary change is necessarily slow, and that there is high genetic similarity between human populations worldwide. I counter-argue that what is necessarily slow is the building-up of complex mechanisms. Undoing this can be much quicker. Moreover, rollback of cognitive mechanisms need not require any genetic change. Finally, I argue that cross-cultural similarity in some trait need not be rooted in genetic similarity. This is not intended as decisive evidence that rollback has occurred. To finish, I suggest ways we might decide whether moral attitudes are likely to be rooted in unchanged Stone Age modules, given that I have argued that cross-cultural similarity is not enough.

## Introduction

According to many Evolutionary Psychology accounts, human moral attitudes are rooted in cognitive modules that are biologically evolved adaptations to conditions in the Pleistocene epoch (e.g. Cosmides and Tooby 1992, 2006; Pinker 1997; Haidt 2003; Krebs 2005; Haidt and Kesebir 2010; Kurzban 2012). These modules evolved as adaptive responses to problems of social interaction—for example, the problem of how to reap the benefits of cooperation and exchange while avoiding the costs of being cheated, or the problem of knowing who to help, who to fight, and when. The key components of this account of the roots of moral attitudes are (i) that cognitive modules evolved in the Pleistocene to deal with such problems; (ii) that these modules have remained largely unchanged since the Pleistocene and (iii) that they continue to underpin our moral attitudes and affect our behaviour today. In the present paper I do not question claim (i), but I question claims (ii) and (iii). There are two alternative scenarios which deserve serious consideration: They may have changed more substantially than standard Evolutionary Psychology allows, thus controverting (ii); or they may no longer play as significant a role in shaping present-day moral attitudes or behaviour as they did in the Pleistocene, thus controverting (iii). Both of these possibilities need to be considered, I argue, because of the extensive role that *outsourcing*, or embedded cognition, plays in the cognitive life and behaviour of modern humans. It can be shown that outsourcing is used in many domains of modern human life, and strong prima facie evidence can be given that the moral is another of those domains. Therefore, I will argue, it is possible that humans do not possess as much internal machinery for guiding actions or for morally responding to situations as the Evolutionary Psychology story alluded to above suggests. Instead, it may be that we often rely on external scaffolding to guide us both in how to act and how to react. I will suggest that this may be the case even in types of situation which our Pleistocene ancestors were provided by natural selection with mechanisms for dealing with. Moreover, it is possible that, because this capacity for outsourcing exists in the modern world, cognitive modules that evolved for dealing with those situations in the Stone Age may no longer exist, or at least may be significantly reduced.

I do not claim that our moral attitudes can be *wholly* explained in the embedded-cognition way that I propose. The main point of my arguments in this paper is that an explanatory factor exists that has not been given much consideration in the literature on evolutionary explanations of moral attitudes. There are, I will argue, at least good prima facie grounds for thinking that this explanatory factor—embedded cognition based on legal institutions—does in fact explain quite a lot of our moral attitudes. Moreover, if and to the degree that this is the case, consequences follow that are importantly different from the consequences that the more usual Evolutionary Psychology explanations of our moral attitudes appear to have.

The structure of this paper will be as follows: In Sect. 2, I will outline the arguments for the usual Evolutionary Psychology approach to explaining our moral attitudes. This includes arguments for why the cognitive modules that those attitudes rest on have not changed since the Pleistocene. Then, in Sect. 3, I will give an account of embedded cognition, and show how legal institutions facilitate a kind of embedded cognition for solving problems of social interaction, thus diminishing the importance of any evolved cognitive mechanisms that there might be for solving social problems. Based on this I will argue that this creates conditions favourable to *evolutionary rollback* of the cognitive modules that, according to Evolutionary Psychology, evolved in the Pleistocene and underlie our moral attitudes. In Sect. 4, I will argue that such evidence as there is for universal moral attitudes can be explained consistently with the previous points. Finally, in Sect. 5 I will make some concluding remarks, briefly outlining some consequences of my view and making some very general suggestions on how my hypothesis might be tested.

Before I proceed, a couple of preliminary remarks are in order. First, I want to emphasise that I am, in this paper, sitting firmly on the fence regarding the issue of whether there is an objective morality, and regarding whether this is knowable by humans. That is not to say that I am invoking the naturalistic fallacy, but simply that I am not in this paper addressing those questions. I am interested in causal explanations of certain aspects of human psychology and behaviour. Second, I am also sitting on the fence with regard to the idea of *extended* cognition. That is, I am taking no side on the issue of whether objects external to the brain, such as calculators or notebooks, count as *part* of the mind, or on the question of whether someone relying on an object external to the brain can be said to be (in the case of a notebook) remembering, or (in the case of a calculator) calculating (Clark and Chalmers 1998). Even if one does not take a stance on these extended cognition claims, one can still accept that people often use external resources to perform tasks which, were they performed internally, would require prodigious feats of memory, calculation etc.

## Evolutionary Psychology and the origins of moral attitudes

Evolutionary Psychology and similar disciplines have a story to tell about the problems of social interaction that our prehistoric ancestors faced—problems such as: who to co-operate with, how to spot cheaters etc.—and about how natural selection is likely to have produced mechanisms to solve them. Many people think that the mechanisms so produced lie at the roots of our moral attitudes today—perhaps not wholly capable of explaining those attitudes all by themselves, but at least playing a major part in their formation (see the list of references in the first paragraph of this paper). By moral attitudes here I mean both moral reactive attitudes—such as moral approval and disapproval, guilt and indignation—and moral motivating attitudes such as the conviction that one ought to do or refrain from doing something for moral reasons. I wish to remain neutral in this paper regarding whether these attitudes should be thought of as emotions or beliefs, or as some mixture of the two, or as belonging to some third category.

The accounts that are the particular target of my critique in the present paper are those that fall within the camp of (capital-E, capital-P) Evolutionary Psychology, rather than (small-e, small-p) evolutionary psychology. I am here adopting a terminological distinction made by David Buller: whereas evolutionary psychology is any study that looks at how the human mind evolved, Evolutionary Psychology has a clearly defined set of underlying assumptions (Buller 2005). The key assumptions of Evolutionary Psychology for present purposes are that the underlying ‘architecture’ of the human mind consists wholly, or almost wholly, of cognitive modules, that these cognitive modules are adaptations, that they evolved in the Pleistocene epoch, and that they are largely unchanged since the Stone Age. I acknowledge that there are evolutionary accounts of human nature that explicitly reject the first of these assumptions (e.g. Sterelny 2012). Such accounts are not targets of my critique in this paper. There are other accounts of the origins of moral attitudes which, though not explicitly based in a modularity view, do not appear to explicitly exclude it, and in any case clearly take them to rest in psychological properties that, they maintain, humans possess more-or-less unchanged since prehistoric times (e.g. Ridley 1996; Skyrms 1996; Joyce 2007). Although I will only mention the fact in passing, such accounts may also be vulnerable to my critique in this paper, assuming my critique is successful. However, the capital-E capital-P version of Evolutionary Psychology is the primary target, and remains a very widely discussed version, having generated not only a large literature of its own but a large literature antagonistic to it as well (e.g. Rose and Rose 2000; Buller 2005; Richardson 2007; Laland and Brown 2011).

Within the literature of Evolutionary Psychology is a significant subset that deals with the origins of moral attitudes (see list of references in the first paragraph of this paper). The key features of Evolutionary Psychology explanations of moral attitudes are that they are underpinned by cognitive modules, that these cognitive modules are adaptations to problems of social interaction that arose in the Pleistocene, and that they have remained largely unchanged since the Pleistocene. It is central to the explanatory project of Evolutionary Psychologists to hold the last of these. For example, Leda Cosmides and John Tooby argue that “[o]ur minds are equipped with moral heuristics that were designed for a small world of relatives, friends, and neighbors, not for cities and nation states of thousands or millions of anonymous people”, and that “our moral heuristics are now operating outside the envelope of environments for which they were designed [i.e. the Pleistocene]” (Cosmides and Tooby 2006, p. 207). This claim is needed by anyone who claims that the biological evolution of our prehistoric ancestors is relevant to understanding why humans have the moral attitudes we do today. Otherwise, the stories about the problems of interaction faced by our ancestors would at best only help us to understand the moral attitudes of our ancestors. Like other evolved traits, these mechanisms are seen by Evolutionary Psychology approaches as having been ‘designed’ by natural selection to maximise reproductive fitness in Pleistocene conditions. To justify the claim that they are unchanged, Evolutionary Psychologists point out that evolutionary change is very slow, and that there is today a general high level of genetic similarity between different human populations all over the world. They also point to similarities between cultures as evidence that there has been no change in underlying mechanisms since the Pleistocene.

For present purposes, I am willing to grant that their account has some plausibility, as an account of what may have happened in the Pleistocene. It is likely that early humans had problems of social interaction of the kind described, which created selection pressure. It is unlikely that they could have been solved efficiently by conscious reasoning, but they could have been solved by cognitive modules. If we grant this, it is plausible that cognitive modules of the kind described by Evolutionary Psychology did evolve in the Pleistocene. I will spell these points out in a little more detail.

### Pleistocene humans faced problems of social interaction

Evolutionary Psychology explanations of where our moral attitudes come from start from the undoubted fact that humans are, and our pre-human ancestors for a long time were, social creatures. This means, among other things, that we both benefit from co-operation with other individuals and suffer when other individuals fail to co-operate. It also means that there is an incentive to take the benefits of others’ co-operation while not reciprocating. For example, hunting is an enterprise that often requires multiple co-operating individuals to be successful, but also often involves those individuals taking risks with their own safety. (Think of hunting a large animal such as a woolly mammoth, with stones and spears, rather than the less risk-taking activities of most hunters today). Assuming these to be the case, I benefit from the fact that others are co-operating in the enterprise, but there is an incentive for me to duck out of its riskier aspects (perhaps by hiding at a crucial moment).

Moreover, and the foregoing example also illustrates this, the co-operators have an incentive to be able to catch and penalise cheaters, in order to avoid losing out through being repeatedly cheated. And if this affects the co-operators’ behaviour, then the cheaters have an incentive to avoid detection. Moreover still, hunters will have different success levels on different days, so that on one day some return to the larger group empty-handed while some return with a large haul, but on a different day it is the other way around. This could create an incentive to share, but only with those whom one can trust to share back. This in turn creates an incentive to be seen as someone who will share, whether one actually is or not. These and other problems, which I am calling problems of social interaction and exchange, require individuals to have some strategies for dealing with them.

### It is unlikely that those problems were solved by conscious reasoning

Evolutionary Psychology accounts of the origins of morality have it that natural selection provided our ancestors with mechanisms for dealing with these problems—mechanisms that switch on automatically in the appropriate circumstances, just as we get hungry automatically when our bodies need food, saving us having to consciously calculate when we need food. Similarly, we often become indignant when we spot cheating, without taking time to calculate whether or not we are harmed by this particular instance of cheating; on other occasions our sympathies are aroused and we help others or cooperate, without calculating whether or not we would be better off in this instance not cooperating. This is because the design of cognitive modules is often a trade-off between accuracy and speed: 100% accuracy is probably impossible, but a mechanism that responds quickly and is right 75% of the time may be more useful than one that responds more slowly and is right 99% of the time.

Not all the evolved mechanisms for dealing with social interaction need be thought of as distinctively moral or even proto-moral. For example, it is plausible that we have evolved mechanisms for reading facial expressions and body language, including for example for signs that a person is telling the truth, or for signs that a person has friendly intentions. But on Evolutionary Psychology accounts we have also evolved feelings of approval or disapproval for certain types of actions, or aversions to doing certain kinds of thing, and we may call these feelings moral, or at least proto-moral. They may, like hunger, arise quickly and without conscious deliberation in the relevant circumstances. But behind them lies complex information-processing, just as in the case of reading facial expressions, and we are usually only consciously aware of the outcome of this processing. This processing may be thought to have a role in guiding behaviour, and (on an optimistic view) in keeping behaviour more-or-less on the straight-and-narrow, morally speaking.

For all this to be any help in understanding how we got to have the moral attitudes we now have, it would have to be the case that whatever the relevant mechanisms evolved in the Pleistocene are, they are largely unchanged and still in some way active in shaping our moral attitudes in the present day. This does not imply that they play any justificatory role, unless we commit ourselves to the view that evolution has implications for how we ought to be, which neither I nor, typically, Evolutionary Psychologists, do. Rather, it implies only that the evolved mechanisms are causally involved in making those present-day attitudes what they are. For example, we may think of our present-day ideas about fairness or equal treatment of equals as in an important way influenced by the same mechanisms as those that cause capuchin monkeys to react violently against unequal treatment. In studies (Brosnan 2006) these monkeys were initially trained to give tokens to human experimenters, and received a piece of cucumber as a reward. The scenario was then changed, with some monkeys receiving a grape—which they apparently value more highly than a piece of cucumber—in exchange for the same token. The ones who received the ‘lesser’ reward were rather unhappy about this, often rejecting the piece of cucumber—for example, throwing it away—even though they had been perfectly happy with a piece of cucumber before. So it seems that their willingness to accept the situation depends on how they see other individuals being treated. In other words, they are unhappy with unequal treatment. As we might put it, perhaps over-anthropomorphising, they feel anger or indignation at being treated unfairly. Similar behaviour has been found in chimpanzees (discussed in de Waal 2013).

There are analogies here with the stories that Evolutionary Psychology tells us about other problems that are evidently solved by cognitive modules, e.g. language learning, face recognition. In all these cases, the standard Evolutionary Psychology position is that there has not been enough time for natural selection to have significantly modified them. This in turn, according to Evolutionary Psychologists, is because they are complex mechanisms, and it takes natural selection a long time to produce complex mechanisms, or to modify them in any significant way while still keeping them functional. This argument is often bolstered by appeal to the comparative lack of genetic diversity between different human groups around the world.

In addition to these general arguments for the unlikelihood of evolution having significantly altered those cognitive modules, there are at least two other arguments that must be considered in any attempt, such as mine, to resist the claim that such cognitive mechanisms must have remained unchanged since the Pleistocene. Firstly, if there is evidence of cross-cultural similarity between groups of people in widely different parts of the world, that seems at least prima facie to support the claim that traits are products of evolution that took place before those groups became separated. Second, there is the phenomenological evidence of immediacy of perception of something as wrong. At least very often, the feeling of moral disapproval or indignation, the feeling that something is *just wrong*, hits us prior to any reflection on why it is wrong (see Haidt 2003). This suggests an analogy with the way colours just hit us, or the way we just recognise people’s faces, and hence that an automatic, unconscious process, i.e. a cognitive module, underlies the perception. I will need to address these arguments in order to defend my alternative proposal, and I will do so in Sect. 4.

## Outsourcing and rollback

The alternative view that I propose rests on the idea of *embedded cognition* – that is, the idea that, very often, our brains are spared the task of performing complex information-processing or storage tasks internally, because there are resources in the external world that obviate the need for those tasks to be so performed (Clark 1997; Haugeland 1998). Although there are examples of this in other parts of the animal kingdom, it does seem that we humans are exceptionally clever at ‘outsourcing’ cognitive work in this way. There are many examples of human technologies that, either by design or as a by-product, save us from having to do cognitive work with our brains. When we use our phones to store phone numbers or other information, or calculators to do sums, we are saving ourselves the trouble of using our brains to perform these tasks. Other cases can involve distributing the cognitive work around multiple different, perhaps non-contiguous, objects. Or, to rephrase this in language that does not presuppose *extended* cognition, they involve savings in cognitive effort being made possible by multiple different, perhaps non-contiguous, objects. These multiple objects may include other agents. For example, Evelyn Tribble (2005) argues that theatrical practices in Shakespeare’s time were designed to minimize the amount that the actors had to commit to memory in order to perform their roles (see also Sutton 2010). A symphony orchestra works on similar principles: the conductor, by giving different individuals and sections their cues to come in on, providing a common beat and so forth, saves the players having to remember or decide these things for themselves. Similarly, it is normal in the natural sciences today for a paper to be credited to multiple authors, reflecting the fact that multiple individuals’ research contributed to the paper’s conclusions, and also in many cases necessitating high levels of trust between the authors of a paper (Claxton 2005). A 2011 paper in *Nuclear Physics B* is credited to approximately 3000 authors (ATLAS Collaboration 2011).^1^

In some cases, our reliance on external cognitive-labour-saving devices can cause the internal mechanisms that we would otherwise use to effectively atrophy, while our skill at using the external resources increases. For example, the psychologists Sparrow et al. (2011) studied the effects on people’s memory of the increasing presence of computers as resources we can use to store and obtain information. One of the things that they found was that when people know that a piece of information is being stored somewhere where they can readily access it, for example in by being told what folder it is in, their ability to remember the information itself decreases, but their ability to remember the relevant folder increases. This suggests that people are quite amenable to making use of external resources to perform or obviate tasks—in this case, the task of remembering—when those resources are available, and that they do so quite naturally without needing any encouragement to do so.

A number of authors have recently proposed that embedded or extended cognition plays a crucial role in our moral reasoning (Scott 2009; Sneddon 2011). The form that this embedded/extended cognition takes for these authors is via our ability to perceive the moral dispositions, feelings, likely responses etc. of the people around us. But these proposals do not mark a very radical departure from the more usual Evolutionary Psychology approaches to explaining moral attitudes, whereby they are underpinned by cognitive modules. I say this because it is consistent with the standard accounts, and it is explicit in some of them, that a crucial part of the causal underpinnings of moral attitudes is quasi-perceptual abilities to read other people’s minds, which in turn rest on evolved cognitive modules evolutionarily ‘designed’ for the purpose of solving the problems of social interaction I discussed earlier (e.g. Dunbar 2004).

While I do not deny that such abilities exist or that they play a role, I believe that the proposals of Scott and Sneddon do not go far enough in acknowledging our capacity to offload the work of moral reasoning. My proposal is somewhat different from theirs: I propose that *legal institutions* can allow us to offload much of the work involved in solving problems of social interaction. By legal institutions I mean those entities and structures, external to the beliefs and habits of individual human beings, by means of which laws are instituted, changed, and enforced. I intend the term to include written documents, such as law codes, contracts, and treaties; I also intend it to include physical structures, such as prisons and courthouses, and formally organised bodies of people, such as police and legal professionals, which bodies are themselves usually created by written documents of some kind. A key defining feature of legal institutions for the purposes of this paper is that they are over and above mere moral (or other) norms—that is to say, over and above moral beliefs, emotions or habits that people have, even large groups of people having the same ones. They have an existence external to people’s mental states, even if (as Searle 1995 argues) their power depends on people’s mental states. They can continue to operate even if the majority of people in a state do not agree with them.

I want to suggest that these institutions can, at least to a large extent, solve precisely those problems that the standard evolutionary accounts described above say that we have evolved cognitive mechanisms to solve. That is, they can solve problems of social interaction and exchange, such as deciding who to co-operate with and who not to, how to react when someone cheats, and so forth. In present-day societies, and in probably every culture known to history, laws have been available as a recourse to some people in at least some situations where they have been mistreated. It must be emphasised that it is very often only *some* people to whom the law offers this protection, and only in *some* situations. In societies where slavery is legal, slaves have usually been denied any such protection. Similarly, in many societies throughout history, women and children have had no legal recourse against being assaulted by their husbands or parents. Note, though, that often in such cases the law not only fails to provide protection, it also prevents the unprotected individuals from carrying out retribution themselves. Slaves cannot punish their masters for bad treatment, and even though there have been slave rebellions in history, such rebellions, being illegal, are difficult.

Thus, whether you are an individual protected by the law or not, legal institutions in many situations either reduce your incentive to punish other individuals who have wronged you or positively disincentivise you from doing so: either they render it unnecessary if you are one of the lucky ones, or they put obstacles in the way of you doing it if you are not. Moreover, if you are one of the protected ones, in many situations if you have been mistreated you do not even have to make a complaint for the miscreant to be liable for punishment. For some crimes, the police and the courts can take action without the victim making any complaint. Similarly, if you are one of the unprotected, the law’s prevention of you from taking action does not depend on you agreeing that you should not.

In the case of capuchin monkeys, individual monkeys respond to unequal treatment by *themselves* becoming agitated, and it is presumably their behaviour when thus agitated that acts as the disincentive against unequally treating them. Their behaviour arising from their own agitated feelings is the monkeys’ only recourse, and it depends on them actually having those agitated feelings: there is no external thing that will become agitated on their behalf or punish others on their behalf. There is some evidence that among some monkeys and apes a group will gang up to punish a misbehaving individual (e.g. the rhesus monkey behaviour described in Hauser 1992), and in such cases the ones doing the ganging-up do not all have to be individuals who have been adversely affected by the misbehaviour. It is currently a matter of scientific controversy whether third-party punishment exists in non-human animals (see Raihani et al. 2012; Riedl et al. 2012). But even if it does, it is different from the punishment that happens by means of legal institutions in an important way. Just as in the capuchin case, the behaviour is not scaffolded in any way by written documents, physical structures, or formally organised bodies. It depends entirely on the activation of angry or indignant responses in the brains of individual monkeys.

But this does not need to be the case if there are laws that give us recourse in situations where we have suffered, or perceive ourselves to have suffered, injustice. Rather than positive imperatives or specific rules, or even intuitions or moral sentiments, the internal, brain-based part involved in all this could be no more than such basic ideas as that *laws are things to be obeyed*, or the knowledge that *if you break the law you will suffer bad consequences*. I admit, however, that it is prima facie unlikely that it is only this. And I must emphasise that I am not claiming that *all* of our moral decision-making is done for us by external resources in this way. However, if a reasonably significant amount of it is, then that reasonably significant amount falls outside the scope of the usual Evolutionary Psychology-based explanations, or indeed any account that takes us to have fundamental moral attitudes, or psychological characteristics underpinning such attitudes, that have remained unchanged since prehistoric times. Moreover, the potential for us to outsource more of our moral decision-making than we currently do is not obviously limited in any way. The experiments of Sparrow et al. suggest that we have the tendency to increase the amount of outsourcing we do when the opportunity presents itself. I am content to argue that a reasonably significant amount, rather than all, of our moral decision-making is outsourced. After all, the actors in Shakespeare’s theatre still had to memorise *some* lines.

I suggest that the presence of legal institutions creates conditions favourable to evolutionary rollback of whatever cognitive modules evolved in the Pleistocene to solve problems of social interaction—i.e. those modules that are alleged to underlie moral attitudes.

Evolutionary rollback (AKA evolutionary streamlining, regressive evolution) is where an organ becomes non-functional and eventually becomes atrophied or disappears altogether.

For example, cave-dwelling fish often lose their eyes and, more generally, vestigial organs often become incapable of performing their former functions. This may happen because of the expensiveness of developing an organ that is not needed. Or there may be costs to having an organ, e.g. risk costs or maintenance costs. Or there may be lack of selection pressure where it is needed to maintain an organ. (For some discussion, see Protas et al. 2007).^2^ What I am suggesting is that even if cognitive modules evolved in the Pleistocene to solve problems of social interaction, conditions since then have been favourable to the rollback of those modules. As Mark Rowlands (1999) argues, in general if a creature no longer benefits from having a complex internal mechanism, then it probably benefits from not having it, a principal reason being the energy costs involved in constructing and maintaining it. Moreover, on what he calls the ‘Barking Dog’ principle, he argues that it makes good evolutionary sense for organisms to make use of external resources when such resources are available to them. This, of course, depends in part on whether the organism is *able* to use them. But we know that laws are available for people to use, and that people are able to use them, to resolve problems of dealing with non-co-operators and so forth.

To sum up my argument so far:

1. Modern humans possess a means for solving problems of social interaction, in the form of legal institutions;
2. Legal institutions can be thought of as facilitating a kind of embedded cognition, often saving us having to rely solely on our own internal cognitive resources to solve these problems;
3. The emergence of legal institutions introduces a new factor in evolution, which leads to the diminution in importance of internal, genetically specified cognitive mechanisms;

This applies to moral attitudes because of the existence of institutions that solve problems of social interaction, saving us having to. Legal institutions often solve those problems that Evolutionary Psychology says we have cognitive mechanisms to solve. Often, a person who has been mistreated does not even have to make a complaint for a miscreant to be liable for punishment. In the case of people who are not so protected, the legal institutions disincentivise them from acting. This can be contrasted with a situation where people’s only response to unfair treatment is to *themselves* become agitated and take punitive action, as appears to be the case with capuchin monkeys.

The conjecture proposed here owes much to Andy Clark’s ‘007’ principle as well as to Rowlands’ ‘Barking Dog’ principle:

In general, evolved creatures will neither store nor process information in costly ways when they can use the structure of the environment and their operations upon it as a convenient stand-in for the information-processing operations concerned. (Clark 1989, p. 64).

If it is necessary for an organism to be able to perform a given adaptive task T, then it is differentially selectively *dis*advantageous for that organism to develop internal mechanisms sufficient for the performance of T when it is possible for the organism to perform T by way of a combination of internal mechanisms and manipulations of the external environment. (Rowlands 1999, p. 80).

Lawrence Shapiro (2010) objects to the 007 and Barking Dog principles on the ground that they make Panglossian assumptions. He argues that both principles require that “regardless of the ancestral condition, evolution will always lead toward a manipulationist strategy” [i.e. one that uses external resources instead of internal ones] (p. 410). In other words, he is arguing that Clark and Rowlands are presupposing an unrealistic view of evolution where what evolves is determined solely by what would be most useful to the organism, and that they do not take seriously the effects of ancestral conditions in biasing the direction of evolution. In response to this I argue, first, that anti-Panglossianism would not favour the standard Evolutionary Psychology story. Any objection to the effect that biases resulting from ancestral conditions are not being taken seriously can be levelled with equal justice at a story that, appealing to evolution, claims that our psychology is underpinned by cognitive modules that were naturally selected in the Pleistocene and hence are well-suited to life in that time. Second, I do not make Panglossian assumptions in the present paper because what I offer, I offer as a conjecture only. I am arguing that there are grounds for thinking that conditions have been favourable to rollback of certain cognitive modules. But that is not the same as claiming that that rollback has taken place. The latter is an empirical question, and at the end of the paper, I make some very brief and tentative suggestions as to how it might be tested. However, regardless of whether or not the 007 or Barking Dog principles are true, we have seen that there is evidence that humans do in fact have a strong tendency to make use of outsourcing opportunities when they are available.

## Response to arguments for the ‘we have not changed’ hypothesis

### Response to the ‘slowness of evolution’ argument

I will here respond to the ‘slowness of evolution’ argument given by Evolutionary Psychologists in support of their claim that cognitive modules evolved in the Pleistocene have not changed. As we have seen, the complexity of the mechanisms is a key component of the argument that it would take a very long time for natural selection to produce them. However, it is a defining feature of embedded cognition solutions that they require *less* internal complexity than ones that involve more ‘onboard computation’. But it is a lot easier for natural selection to un-make a complex mechanism than it is for it to make it. Following the suggestions of Clark and Rowlands, I suggest that work that might at one point in humans’ evolutionary history have been done internally (‘onboard’) can come to be done externally (‘offloaded’ or ‘outsourced’), thus allowing a saving of energy through evolutionary rollback.

Rolling back, since it only involves un-making a mechanism, can be much faster than building one, since it is a much simpler achievement. It only requires natural selection hitting upon a way that the mechanism no longer works, and there are plenty of those. One only has to reflect on how much easier it is to break a complicated machine than it is to make one to see this. It may be the case that humans at one time had internal, developmentally robust moral or proto-moral attitudes, and that that was the only way to deal with social interaction issues at the time. However, most or perhaps all human groups now existing have legal institutions of some kind, in the sense spelt out in Sect. 2, that can at least in some cases serve that function externally. Any genetic change needed to facilitate a switch from relying on internal resources to external ones would, in all likelihood, have been very simple and hence could have been brought about by natural selection quite quickly. But I see no reason why it could not have happened without any genetic change at all, through humans’ development following a new pathway due to changes in the environment. In this case, the relevant change in the environment is the increased opportunities for humans to outsource cognitive tasks, coupled with humans’ tendency to use those opportunities when they are available.

Of course legal institutions are also complex. However, they are no more complex than other human-made entities which have clearly come into existence later than the Pleistocene, and which therefore cannot have required genetic change to come into existence. For example, the infrastructure of a modern city is vastly more complex than anything that existed in the Pleistocene, or for that matter in ancient historical times, but did not, so far as we know, require genetic change to come into existence.

Thus, rather than positive imperatives or specific rules, or even intuitions or moral sentiments, what is left of our evolutionary heritage from the Pleistocene may be no more than such basic ideas as that laws are things to be obeyed. To reiterate, I do not think it is likely that it is *that* minimal, but it is an empirical question whether and to what extent the rollback about which I am hypothesising has taken place.

### Response to the ‘cross-cultural similarity’ argument

In this subsection I will argue that evidence of cross-cultural similarity does not necessarily support the standard Evolutionary Psychology view, since there is an alternative explanation for such similarity consistent with the conjecture offered here. That alternative explanation is that laws were made in a given society because somebody in that society thought they were a good idea. And sometimes the same thing is a good idea in lots of different cultures.

It is possible for advocates of the standard Evolutionary Psychology view to appeal to any direct evidence that there might be that there are moral values that are common to all or most cultures. As with evolutionary theories about human mating preferences and other things, it is especially important to look for evidence from different cultures that have had no contact with each other. In practice, this means (or at least ought to mean) looking for evidence from cultures that have not had contact with modern western culture, in order to alleviate the suspicion that the values of modern western culture are being projected onto humanity in general. The debate about whether there is pan-cultural agreement on fundamental morals is a very old one. In Book I, Chaper III of the *Essay Concerning Human Understanding*, Locke argued that there was no such agreement, but in the corresponding chapter of his response to Locke, *New Essays on Human Understanding*, Leibniz argued that there was. Lewis, in *The Abolition of Man* (1943), offered an ambitious list of moral rules that he claimed could be found in all known cultures. A recent survey paper by Henrich et al. (2010) suggests that traits commonly taken to be universal by people from “Western, Educated, Industrialized, Rich, and Democratic (WEIRD) societies” (p. 61) are in fact highly unusual.

I do not propose to adjudicate the matter here, but the claim that there is widespread pan-cultural agreement on moral matters is—to say the least—contentious, and this contentiousness is not due solely to lack of evidence but also to the difficulty of interpreting such evidence as there is. However, let us say for the sake of argument that there is good evidence that some moral values are constant across many different unconnected cultures. That would tell against any explanation of those values in terms of cultural transmission. That, in turn, might suggest that the only explanation remaining is that we inherited those values—or at least the proto-values, emotional reactions or whatnot—underlying them, from a time at least as long ago as the last common ancestor. And that, in turn, might suggest that the only plausible explanation for them is in the evolution of our Pleistocene ancestors.

But, insofar as there are such universals, an obvious alternative explanation is that some rules of conduct are a good idea in lots of different circumstances. All that is required to explain why something is legally prohibited or legally required is that somebody somewhere thought it would be a good idea to prohibit or require it. But it is perfectly possible that some things—murder, for example—are such that people in many different societies would find it a good idea to make laws against them. Of course, what is counted as murder can vary greatly—e.g. some people consider abortion murder, others do not; some people consider killing animals murder, others do not; some people consider war murder, others do not. However, what I am addressing in this subsection is how to explain such commonalities as there are: the existence of major differences in moral beliefs between cultures or between individuals does not favour the view that our moral attitudes are to be explained by a universal set of cognitive modules. Other things—enforcing certain types of promises, for a possible example—may be such that many different kinds of societies would find it a good idea to introduce laws instituting them. So what I am proposing is that laws were invented in a given society because somebody thought they were a good idea, and in many cases what is a good idea is invariant across cultures, and this in turn because they solved some problem that people reasonably regularly face when interacting with each other.

The issue of explaining why we find similar laws and customs in many different societies is approached in a standard Evolutionary Psychology way by Boyer and Petersen (2012). They argue that the commonalities reflect the fact that humans find certain types of social arrangement ‘natural’, because of evolved cognitive modules that were adaptive for our Pleistocene ancestors. This view places much less weight than mine on the conscious element in law-making. If the type of story Boyer and Petersen offer is true (and they do say that it is only true of ‘many’ institutions, not all), lawmakers do not reflect on why a law is a good idea. Rather, lawmakers find certain laws just ‘right’, as a result of unconscious operations of cognitive modules. Such an explanation seems to me to greatly underestimate the amount of conscious thought that goes into law-making. Very large parts of the law—e.g. constitutions (with the exception of Great Britain’s ‘unwritten constitution’), parliamentary statutes, treaties—are codified and written down, after a process of conscious deliberation. This deliberation does not have to be by just one person: it can be done by groups—e.g. by committees, negotiating teams, or parliaments. However, I do not deny that *in some cases* a story of the Boyer and Petersen type may be correct, and I will say a little in the final section about how in individual cases we might tell if this is so, or if my type of story is correct instead.

Also at odds with the explanation I have just suggested is the idea of ‘cultural evolution’ as developed in the work of—for example-Boyd and Richerson (1985). They argue that cultural phenomena (such as languages, customs, fashions, styles of art, religion) evolve by a process analogous to biological natural selection. This would imply that they are not products of conscious design. I do not deny that this may apply to some aspects of the law: we may sometimes speak of a certain area of the law ‘evolving’, perhaps as wider cultural attitudes evolve—e.g. in the matter of sexual mores—and this may be by a process analogous to natural selection. But in response to this I would say: firstly, that even if this is the case, it does not affect the main point of the present paper—namely, that the presence of robust legal institutions can facilitate cognitive outsourcing and rollback. Second, an overemphasis on the unconscious nature of cultural evolution would once again underestimate the amount of conscious thought that goes into law-making.

It may be objected that I have only offered an extremely vague explanation for such commonalities of legal and moral systems as may exist. Theoreticians trying to construct a genuinely informative account of how moral value-systems evolved could end up arguing forever about what exactly are the rules that many different societies are likely to have thought were a good idea. However, I suggest that it is at least as good as common-inheritance evolutionary explanations of moral universals, since the latter suffer from this problem to at least as great a degree. That is because they take the reason why these moral attitudes exist to be that they solved Pleistocene problems of social interaction. Yet a great many of the Pleistocene problems of social interaction hypothesised by Evolutionary Psychology and similar schools are of such a general kind that they are common to any human society, Pleistocene or not. More generally, accounts of the problems faced by our Pleistocene ancestors tend to be rather vague as a consequence of the paucity of our knowledge of Pleistocene conditions.

### Response to the phenomenological argument

As I said earlier, some plausibility is given to view that our moral attitudes are grounded in cognitive modules by the fact that, very often, the feeling of moral disapproval or indignation, the feeling that something is *just wrong*, hits us prior to any reflection on why it is wrong. This suggests an analogy with the way colours just hit us, or the way we just recognise people’s faces, and hence that an automatic, unconscious process, i.e. a cognitive module, underlies the perception. If we find this plausible, then we can combine this with the complexity of the processes that likely underlie this to argue that they are likely to be products of natural selection.

However, people seem to have these immediate visceral responses to very different kinds of things. Some people have such responses to swear-words, some to racist or homophobic language, while others are unbothered by such things. In saying this, I am not claiming that there are no moral universals, but rather that the kind of immediate visceral moral-indignation response that forms the basis of a phenomenological argument for there being cognitive modules underlying our moral responses, seems at least prima facie to be evocable by some things that are clearly not moral universals. (I will say a little bit more about this at the end of the paper).

This point highlights a strength of my view. The standard Evolutionary Psychology story requires us to explain modern moral attitudes as products of cognitive modules that have remained unchanged since the Pleistocene. But then we have to explain very different moral attitudes as underpinned by the same modules. It is evident from history that what arouses people’s moral approval or disapproval has changed greatly over time. At one time—and a time that is a blink of an eye ago in evolutionary terms—ancestors of citizens of today’s liberal democracies accepted slavery, the prohibition on married women owning property or entering professions, the divine right of kings, etc. etc.

Some attempts to accommodate these facts into to a standard inheritance story have been made (e.g. in Pinker 1997), and a story of the kind offered by Dunbar, Scott and Sneddon has less difficulty accommodating it. But an alternative is to say that our underlying moral attitudes are more changeable than the standard Evolutionary Psychology story allows. There is evidence that moral attitudes such as disapproval can be triggered very easily. For example, arousal of disgust can cause people to form negative moral judgements (Wheatley and Haidt 2005; Schaller and Park 2011). The presence of a bad smell or being in a messy, dirty room increases the likelihood of forming a negative moral judgement on someone, even when the smell or the dirt have nothing to do with them, and even if the person being judged is not present but just being talked about. That people’s moral feelings are triggered in a specific situation is not a reliable indicator that they would on a different occasion be similarly triggered by the same scenario, as opposed to by extraneous factors.

## Concluding remarks

It is worthwhile pointing out that I am saying more than just that our moral attitudes and practices are, at least to a greater extent than Evolutionary Psychology supposes, products of our cultural milieu. That would be consistent with the view that we have highly robust internal moral attitudes, but that those attitudes are products of learning and socialisation in early life, and hence can vary greatly between cultures. This view is held both by people who reject the relevance of biological evolution to understanding human moral attitudes at all (e.g. most of the authors in Rose and Rose 2000), but also by those who think that interesting evolutionary accounts can be given of how humans come to have the capacity to be so influenced by culture, and of what mechanisms underpin that capacity (e.g. Wright 1994; Sterelny 2012). The hypothesis I am suggesting differs from theirs in that I think that, at least to the extent that legal institutions have either facilitated the offloading of moral-cognition tasks, or prevented people from effectively exercising their moral capacities, it may be that robust internal moral attitudes do not exist at all, or at least exist to much less an extent than we commonly think: our moral attitudes, or at least a significant amount of them, are momentary and are the effects of immediate conditions.

This has some parallels with an idea explored by Gilbert Harman (1999, 2000) and Doris (2002), who argue based on social psychology that there is no such thing as character as it is commonly understood. It seems to me that there is indirect evidence that supports the embedded cognition view as against the learning (in the sense of internalisation) view. The evidence from Sparrow et al. cited above suggests that people are very amenable indeed to letting external systems do the work when they know that that option is available. Well-known psychological experiments such as the Stanford Prison Experiment (Haney et al. 1973) suggest that our individual moral beliefs or sentiments are not as robust as would be the case if they were stored in our brains long-term (see Ross and Nisbet 2011 for extensive discussion of this and many other examples).

None of this is intended as decisive evidence that rollback of cognitive modules involved in moral attitudes has taken place. Evidence for the non-robustness or otherwise of our moral attitudes comes from work such as Ross and Nisbett’s, which admittedly still attracts some controversy. I further suggest that there are some ways in which we might be able to tell whether particular attitudes are likely to be rooted in unchanged Pleistocene modules or not, given that I have argued that cross-cultural similarity is insufficient evidence. I offer two suggestions: (1) We might find good evidence that some attitude or practice is pan-cultural and is not explainable as obviously a good idea in lots of different contexts. (2) We might find consistent phenomenological and/or behavioural differences between moral attitudes that are *indisputably* products of culture and ones that are not clearly so.

The alternative account that I offer should help to combat some of the morally pessimistic or quietistic implications that Evolutionary Psychology accounts of morality appear to have. On the usual Evolutionary Psychology accounts, what keeps cognitive modules unchanged is alleged to be a combination of lack of genetic change since the Pleistocene and a very high level of developmental robustness, which implies that only genetic engineering or highly structured and scientifically well-informed intervention in the developmental process could change them. The upshot of this is that we may be stuck with them, even if they conflict with other, perhaps more reasoned, moral beliefs. But the solutions that the standard Evolutionary Psychology type of story envisages natural selection as having produced tend to be ones that worked in the Pleistocene environment, and do not necessarily work at all as well in the present day. In the Pleistocene, a good rule of thumb to follow for eating would be ‘get as much sweet food as you can get’, but today that would positively harmful (Symons 1992).

More generally, the fact that the cognitive mechanisms affecting our moral attitudes have been ‘designed’ by natural selection in the Pleistocene implies that they are not necessarily geared towards our flourishing or happiness in the present day, for two reasons: firstly, because Pleistocene conditions were very different from those of today, and secondly because what maximises reproductive fitness need not at all contribute to flourishing or happiness. Moreover, they need not even have worked perfectly in the Pleistocene, in the sense of always yielding the right answer to a question such as ‘is this person likely to co-operate, or cheat?’ The alternative account that I offer does not have these pessimistic implications. It is possible that some of the implications that it does have are equally worrying, however.

The hypothesis I am proposing differs in its consequences from the usual evolutionary type of story in other ways too. Very roughly, the usual type of story predicts that what constitutes a solution to the problems is a strategy that *maximises reproductive fitness*, so that that is what we should expect to have been produced. My alternative predicts that what constitutes a solution is something that eliminates or at least reduces (or would be expected to reduce) what someone perceives as a problem. Hence there is, at least in principle, the possibility of empirical evidence being forthcoming that would favour one possibility over the other.

## Footnotes

1 I owe this information to Goldstein (2011).

2 Richardson (2007), pp. 117–124 discusses regressive evolution in the context of a critique of Evolutionary Psychology, but does not link it to outsourcing of cognitive tasks as I am doing here. Therefore, the nearest precedents for my argument here are Clark (1989) and Rowlands (1999).

## References

[CR1] ATLAS Collaboration (2011). Measurement of the differential cross-sections of inclusive, prompt and non-prompt J/ψ production in proton–proton collisions at √*s* = 7 TeV. Nuclear Physics B.

[CR2] Boyd R, Richerson PJ (1985). Culture and the Evolutionary Process.

[CR3] Boyer P, Petersen MB (2012). The naturalness of (Many) social institutions: Evolved cognition as their foundation. Journal of Institutional Economics.

[CR4] Brosnan SF (2006). Nonhuman Species’ reactions to inequity and their implications for fairness. Social Justice Research.

[CR5] Buller D (2005). Adapting minds: Evolutionary psychology and the persistent quest for human nature.

[CR6] Clark A (1989). Microcognition.

[CR7] Clark A (1997). Being there: Putting brain, body and world together again.

[CR8] Clark A, Chalmers D (1998). The extended mind. Analysis.

[CR9] Claxton LD (2005). Scientific authorship: Part 2. History, recurring issues, practices, and guidelines. Mutation Research.

[CR10] Cosmides L, Tooby J, Barkow C, Tooby J (1992). Cogntive Adaptations for Social Exchange. The adapted mind: Evolutionary psychology and the generation of culture.

[CR11] Cosmides L, Tooby J, Gigerenzer G, Engel C (2006). Evolutionary psychology, moral heuristics and the law. Heuristics and the Law.

[CR12] de Waal F (2013). The Bonobo and the Atheist: In search of humanism among the primates.

[CR13] Doris JM (2002). Lack of character: Personality and moral behavior.

[CR14] Dunbar R (2004). The human story: A new history of mankind’s evolution.

[CR15] Goldstein, A. (2011). Too many authors. *Berkeley Science Review*, June 6 2011. http://berkeleysciencereview.com/too-many-authors/. Accessed 12 Mar 2018.

[CR16] Haidt J, Davidson RJ, Scherer KR, Goldsmith HH (2003). The moral emotions. Handbook of affective sciences.

[CR17] Haidt J, Kesebir S, Fiske ST, Gilbert DT, Lindzey G (2010). Morality. Handbook of social psychology.

[CR18] Haney C, Banks WC, Zimbardo PG (1973). A study of prisoners and guards in a simulated prison. Naval Research Review.

[CR19] Harman, G. (1999). Moral philosophy meets social psychology: Virtue ethics and the fundamental attribution error. In *Proceedings of the Aristotelian society*, New Series (Vol. 99).

[CR20] Harman, G. (2000). The nonexistence of character traits. In *Proceedings of the Aristotelian society*, New Series (Vol. 100).

[CR21] Haugeland J, Hagueland J (1998). Mind embodied and mind embedded. Having thought: Essays in the metaphysics of mind.

[CR50] Hauser MD (1992). Costs of deception: Cheaters are punished in rhesus monkeys (Macaca mulatta). Proceedings of the National Academy of Sciences.

[CR22] Henrich J, Heine SJ, Norenzayan A (2010). The weirdest people in the world?. Behavioral and Brain Sciences.

[CR23] Joyce R (2007). The evolution of morality.

[CR24] Krebs D, Buss D (2005). The evolution of morality. The handbook of evolutionary psychology.

[CR25] Kurzban R (2012). Why everyone (Else) is a hypocrite: Evolution and the modular mind.

[CR26] Laland KN, Brown GR (2011). Sense and nonsense: Evolutionary perspectives on human behaviour.

[CR27] Lewis CS (1943). The abolition of man.

[CR28] Pinker S (1997). How the mind works.

[CR29] Protas M, Conrad M, Gross JB, Tabin C, Borowsky R (2007). Regressive evolution in the Mexican Cave Tetra, Astyanax mexicanus. Current Biology.

[CR30] Raihani NJ, Thornton A, Bshary R (2012). Punishment and cooperation in nature. Trends in Ecology and Evolution.

[CR31] Richardson RC (2007). Evolutionary psychology as maladapted psychology.

[CR32] Ridley M (1996). The origins of virtue.

[CR33] Riedl K, Jensen K, Call J, Tomasello M (2012). No third-party punishment in chimpanzees. Proceedings of the national academy of sciences.

[CR34] Rose H, Rose S (2000). Alas, poor Darwin: Arguments against evolutionary psychology.

[CR35] Ross L, Nisbet RE (2011). The person and the situation.

[CR36] Rowlands M, Rowlands M (1999). Evolution and Environmentalism. The body in mind: Understanding cognitive processes.

[CR37] Schaller M, Park JH (2011). The behavioral immune system (and Why it matters). Current Directions in Psychological Science.

[CR38] Scott, T. J. (2009): *The evolution of moral cognition.* PhD Thesis, Victoria University of Wellington.

[CR39] Searle JR (1995). The construction of social reality.

[CR40] Shapiro L (2010). James bond and the barking dog: Evolution and extended cognition. Philosophy of Science.

[CR41] Skyrms B (1996). Evolution of the social contract.

[CR42] Sneddon A (2011). Like-minded: Externalism and moral psychology.

[CR43] Sparrow B, Liu J, Wegner DM (2011). Google effects on memory: Cognitive consequences of having information at our fingertips. Science.

[CR44] Sterelny K (2012). The evolved apprentice: How evolution made humans unique.

[CR45] Sutton J, Menary R (2010). Exograms and interdisciplinarity: History, the extended mind and the civilizing process. the Extended Mind.

[CR46] Symons D, Barkow C, Tooby J (1992). On the use and misuse of darwinism in the study of human behavior. The adapted mind: Evolutionary psychology and the generation of culture.

[CR47] Tribble E (2005). Distributing cognition in the globe. Shakespeare Quarterly.

[CR48] Wheatley T, Haidt J (2005). Hypnotic disgust makes moral judgments more severe. Psychological Science.

[CR51] Wright R (1994). The moral animal.

